# Differential regulation of sFlt-1 splicing by U2AF65 and JMJD6 in placental-derived and endothelial cells

**DOI:** 10.1042/BSR20193252

**Published:** 2020-02-25

**Authors:** Adrian C. Eddy, Heather Chapman, David T. Brown, Eric M. George

**Affiliations:** 1 Department of Physiology and Biophysics, University of Mississippi Medical Center, Jackson, MS, U.S.A.; 2 Department of Cell and Molecular Biology, University of Mississippi Medical Center, Jackson, MS, U.S.A.

**Keywords:** alternative splicing, JMJD6, preeclampsia, sFlt-1, U2AF65

## Abstract

Despite years of study, the gestational disorder preeclampsia (PE) remains poorly understood. One proposed mechanism of PE development is increased soluble VEGF receptor-1 (sFlt-1), ultimately causing angiogenic imbalance and endothelial dysfunction. The soluble protein is an alternative splice variant of FLT1, which also encodes for the full-length receptor Flt-1. The mechanism of the alternative splicing, and the reason for its inappropriate increase in preeclampsia, is not well understood. U2 auxiliary factor 65 (U2AF65) and jumonji C domain-containing protein 6 (JMJD6) have been implicated in the splicing of sFlt-1. Using siRNA knockdown and plasmid overexpression in immortalized placental trophoblasts (BeWo) and primary endothelial cells (HUVECs), we examined the role these proteins play in production of sFlt-1. Our results showed that U2AF65 has little, if any, effect on sFlt-1 splicing, and JMJD6 may enhance sFlt-1 splicing, but is not necessary for splicing to occur. Utilizing a hypoxic environment to mimic conditions of the preeclamptic placenta, as well as examining placentae in the reduced uterine perfusion pressure (RUPP) model of PE, which exhibits increased circulating sFlt-1, we found increased expression of JMJD6 in both hypoxic cells and placental tissue. Additionally, we observed a potential role for U2AF65 and JMJD6 to regulate the extracellular matrix enzyme heparanase, which may be involved in the release of sFlt-1 protein from the extracellular matrix. It will be important to study the role of these proteins in different tissues in the future, as changes in expression had differential effects on sFlt-1 splicing in the different cell types studied here.

## Introduction

Preeclampsia is an obstetric disorder that is characterized by new onset hypertension. This disease, affecting approximately 2–8% of pregnancies, remains a leading cause of maternal and fetal morbidity and mortality as well as a leading cause of preterm birth [1]. Though the etiology of preeclampsia has yet to be elucidated, it is believed that impaired placentation occurs, promoting an ischemic and hypoxic placental environment [2,3]. In response to the adverse environment, the placenta will release several factors into the maternal circulation, leading to the development of the maternal syndrome [2]. One highly recognized factor increased in preeclampsia is the soluble vascular endothelial growth factor (VEGF) receptor sFlt-1 [4].

While sFlt-1 is increased during normal pregnancy, the expression of the soluble receptor increases disproportionately in many cases of preeclampsia [5]. In the maternal circulation, sFlt-1 acts as a decoy receptor, leading to the binding of VEGF and its close relative placental growth factor (PlGF), creating an anti-angiogenic environment. Because of the limited biological activity of VEGF and PlGF, endothelial dysfunction develops, contributing to the vasoconstriction of vessels throughout the periphery and renal circulation, and ultimately hypertension [4]. The role of sFlt-1 in preeclampsia has further been demonstrated in studies of sFlt-1 administration in pregnant rodents that result in a preeclamptic phenotype [6–8], and extracorporeal removal of sFlt-1 from the maternal circulation by apheresis during preeclampsia has been suggested to prolong gestation [9].

Though the increase in sFlt-1 expression has been repeatedly shown in preeclampsia, the mechanism for increased sFlt-1 is not fully understood. The soluble receptor represents an alternative splice variant of the transmembrane receptor Flt-1, both arising from the FLT1 gene [10]. Though commonly referred to as a single entity, four splice variants of sFlt-1 with varying C-termini are present in the human placenta. The two most common variants observed are sFlt-1 13i and sFlt-1 e15a [11]. Alternative splicing describes a process in which introns are removed from pre-mRNA, and the exons are reconnected. During this mechanism, alternative exons from the full-length gene are expressed, producing proteins of differing primary sequence from the same gene [12]. The splicing event is catalyzed by a group of small nuclear ribonucleoproteins (snRNP) composing the backbone of the spliceosome. The snRNPs of the spliceosome include: U1, U2, U4, U5 and U6, which all have distinct functions in the splicing process. U2 binds the splice sites at the 3′ end [13,14]. To assist in this role, U2 has several accessory proteins, including U2 auxiliary factor 2 (U2AF2 also known as U2AF65) [12].

Due to its role in splicing, U2AF65 has been implicated in the regulation of sFlt-1 splicing. Additionally, jumonji C domain containing protein 6 (JMJD6) is also believed to be important in the splicing event for sFlt-1. Previous studies have suggested that U2AF65 promotes the splicing event to sFlt-1, while JMJD6 down-regulates this process by marking U2AF65 for degradation [15]. Other studies, however, have demonstrated increased JMJD6 expression in hypoxia and PE [16], and U2AF65 and JMJD6 typically work in the same direction on proteins in which they co-regulate [17]. Because JMJD6 is described as a lysyl-hydroxylase whose function is dependent on oxygen [18], it is unclear if potentially increased expression of JMJD6 would negatively correlate with U2AF65 expression in the hypoxic placenta. Given the role of sFlt-1 in the development of the maternal syndrome mentioned above, it is necessary to develop a better understanding of the splicing event leading to increased sFlt-1 and the proteins involved in this process. Here, we tested the hypothesis that both U2AF65 and JMJD6 are necessary for increased circulating levels of sFlt-1, either by alternative splicing of the FLT1 gene to sFlt-1 or by regulation of extracellular matrix enzyme heparanase, which solubilizes sFlt-1 bound to the extracellular matrix. To accomplish this task, we utilized endothelial cells and placental trophoblasts to perform siRNA knockdown and plasmid overexpression of U2AF65 and JMJD6, as well as assessing the expression of these proteins under conditions of hypoxia and placental insufficiency in the reduced uterine perfusion pressure (RUPP) model of PE.

## Materials and methods

Cell culture: Human umbilical vein endothelial cells (HUVECs) (ATCC; Monassas, VA) as well as the choriocarcinoma placental trophoblast derived cell line BeWo (Sigma) were both used to examine the role of U2AF65 and JMJD6. Cells were grown in either 6-well (HUVECs) or 24-well (BeWo) plates at 37°C with 5% CO_2_ and room-air oxygen levels. Cells were plated with complete media and allowed to settle for 24 h before any treatment began. Hypoxia experiments were performed at 8% and 1% oxygen to mimic normal and preeclamptic pregnancy, respectively. Media were pre-treated in 8% and 1% oxygen for a minimum of 8 h prior to being added to the cells, and the cells placed in the respective incubator for 24 h.

Cell transfections: In order to knockdown expression of the two proteins, siRNA SMARTpools containing multiple specific siRNAs were used for U2AF65 (NM_001012478, NM_007279, XM_006722994, XM_011526410) and JMJD6 (NM_001081461, NM_015167, XR_001752456, XR_001752457, XR_934428, XR_934429) (Dharmacon; Lafayette, CO). Additionally, a negative control containing scrambled siRNA was used (Dharmacon; Lafayette, CO). siRNAs were transfected via liposomal Dharamafect transfection reagent 4 (Dhamacon; Lafayette, CO) with Opti-MEM per manufacturer’s protocol. In order to overexpress U2AF65 and JMJD6, two plasmids were used. Human U2AF65 (NM_007279) was cloned into pCMV6-Entry vector (Origene). Plasmid p6352 MSCV-CMV-CMV-Flag-HA-JMJD6 was a gift from Peter Howley (Harvard University) (Addgene plasmid # 31358; http://n2t.net/addgene:31358; RRID:Addgene_31358). *In vitro* mutagenesis was used to insert the coding region of JMJD6 (lacking the Flag and HA epitopes) into a plasmid expression vector under control of the human CMV immediate early promoter.

The transfection was done using Lipofectamine 2000 (Invitrogen; Carlsbad, CA) per manufacturer’s protocol. Control samples were treated with the Lipofectamine 2000 in the absence of DNA. For both overexpression and knockdown, the transfection was incubated for 24 h before sample collection.

PCR: In order to perform real-time quantitative PCR, cell samples were collected by trypsinization 24 h after the transfection. Cell pellets were immersed in RNALater (Ambion; Carlsbad, CA) for 4 days at 4°C before RNA isolation was performed. To isolate RNA from the cells, the PureLink RNA mini kit (Ambion; Carlsbad, CA) was utilized with its associated protocol. Upon completion of RNA isolation, the concentration of the RNA was measured by Nanodrop 2000c (Thermo scientific; Rochester, NY). The RNA was converted into cDNA using the First Strand cDNA synthesis kit (Thermo scientific; Rochester, NY). Enough RNA to make 300 ng of cDNA was used and the protocol was used as specified. cDNA was stored at −20°C until PCR was performed.

In order to test the expression levels of splicing factors and ECM remodeling proteins, Taqman primers for human mRNA were utilized in conjunction with nuclease-free water and Hot Start Taq 2x Master Mix (New England BioLabs; Ipswich, MA). The following genes were analyzed via Taqman primers: U2AF65, JMJD6, HPSE1, MMP9, and β-ACT (Thermo scientific; Rochester, NY). Samples were run using a C1000 Touch Thermal Cycler and corresponding CFX96 Optics Module Real-Time system head (Bio-Rad; Hercules, CA). To calculate the fold change in expression, the ΔΔ*C*_t_ method was used, normalizing each sample to its own Actin expression and the mean change of the control samples [19]. We also designed our own SYBR Green-based primers for full-length Flt-1 and different variants of sFlt-1, listed below (Invitrogen; Carlsbad, CA). The two variants we measured, sFlt-1 i13 (V2) and sFlt-1 e15a (V3), have been described as being the most commonly observed in the human placenta [11]. The most recent reference sequences for the individual transcripts (NM_002019.4, NM_001159920.2, and NM_001160030.2, respectively) were used to design SYBR-based primers for each with Primer-BLAST (NCBI). Individual primers sets were used to perform end-point PCR (40 cycles) on total BeWo RNA. All primers produced single band products, and amplicon size was verified as correct by running on 1% agarose gels against calibrated DNA standards. SYBR green master mix (Thermo scientific; Rochester, NY) was used for these designed oligo primers. The PCR cycles were the same for both Taqman and SYBR; however, oligo primers with SYBR mix runs also contained a melting curve in order to ensure single product production. Calculations for gene expression were performed the same with SYBR as with Taqman. In order to validate the primers, PCR products were run on an agarose gel to ensure the predicted base pair weight was observed, as well as a single band indicating a single product (Figure 6).

**Table:** 

FLT1 (Full length)
Forward: GAAATCACCTACGTGCCGGA
Reverse: AGAGCTTTGTACTCGCTGGC
sFlt-1 i13 (V2)
Forward: TGGGGAGGGGAGGATGTTAG
Reverse: TAAGGGAGGTGCGTTGAACC
sFlt-1 e15a (V3)
Forward: CGAGCCTCAGATCACTTGGTT
Reverse: GTCTTGGCTCTCCAACTAAAGG

Animal: Sprague-Dawley timed pregnant rats (Charles River Laboratories) arrived at The University of Mississippi Medical Center on the 11th day of gestation. The procedures performed were approved by the Institutional Animal Care and Use Committee of the University of Mississippi Medical Center (Protocol 1403A). They also were in accordance with the National Institutes of Health Guidelines for the Care and Use of Laboratory Animals. The rats were kept on a 12:12-h light and dark schedule at a constant temperature of 23°C and given food and water *ad libitum*.

Reduced Uterine Perfusion Pressure: On day 14 of gestation (GD 14), the reduced uterine perfusion pressure (RUPP) procedure was done, restricting blood flow to the aortic and bilateral ovarian arteries, as previously described [20]. In short, animals received anesthesia via regulated 3% isoflurane (Henry Schein Animal Health; Dublin, OH), and an incision was made along the midline of the abdomen. The uterine horns were both externalized, and then a single .203 mm silver surgical clip was planted on the abdominal aorta superior to the bifurcation of the iliac. To prevent compensatory blood flow, a .100 mm silver surgical clip was placed on the left and right ovarian arteries that supply the uterus. Sham animals were incised, and vessels isolated as above, but no clips were placed. Additionally, mean arterial pressure measurements were performed on GD 19 via carotid catheter indwelling, as previously described [21] (data not shown). Animals were subsequently euthanized by isoflurane overdose, pneumothorax, and cardiac excision.

Tissue collection: on GD 19, rats were anesthetized as stated above. A ventral midline incision was made, and the uterus was externalized. After cardiac excision, the number of both viable and reabsorbed pups, as determined by gross morphology, was recorded in the uterus and the individual pups and placental weights were additionally recorded. Samples of placentas from each uterine horn were obtained and flash frozen in liquid nitrogen followed by storage at −80°C.

Western blot: BeWo cell lysates were used in order to confirm the protein expression seen in the PCR. RIPA (Chem Cruz; Dallas, TX) with its associated protease inhibitors was used in order to collect cell lysates, and the protocol was used as described. Briefly, iced PBS was used to wash the wells 2–3 times and aspirated. Then, RIPA buffer was made and added to the cell wells. The plates were moved to 4°C for approximately 30 min before collecting the cells. The tubes were then spun for 10 min at 4°C and 12000 rpm. The aqueous layer was transferred to a new tube, which would contain the desired proteins. Due to low protein yields in HUVEC cells, these samples were cultured in T25 flasks and cell samples were harvested via trypsin. The trypsin was removed after centrifugation and the cell pellets washed three times with PBS. Cell pellets were then resuspended in RIPA buffer and allowed to sit at 4°C for approximately 30 min before centrifugation and collection of lysates. Similarly, RIPA was used to obtain protein from placental samples. Approximately 100 mg of placental tissue was placed in a Lysing Matrix D tube (MP Bio; Santa Ana, CA) with 1 ml of RIPA. The tubes were homogenized using the MP Bio FastPrep-24 (MP Bio; Santa Ana, CA). The liquid was removed from the tubes, centrifuged for 10 min at 4°C and 12,000 rpm in order to remove any remaining tissue. The fluid was then collected and stored in a fresh tube at −20°C. BCA assay was done in order to determine the total protein concentration of the lysates, and this concentration was used to load approximately the same amount of total protein into the Western blot gel. Similarly, Western blot of serum-less cell media (in these cases, serum-less media were used following the transfection to prevent albumin from impeding the heparanase target) were performed in order to examine protein secreted into the media. For these samples, the same amount of media was loaded into each well.

The samples with 4× loading dye (BioRad; Hercules, CA) were denatured by placement in a heat block at 100°C for approximately 10 min. The samples, as well as protein ladder (BioRad; Hercules, CA), were loaded into a Mini-Protean pre-cast gels (BioRad; Hercules, CA) and run at 200 V for 45 min or until the loading dye was observed at the bottom of the gel. Wet transfer onto a nitrocellulose membrane was performed at 100 V for 45–60 min. The membrane was blocked for 1 h using Odyssey blocking buffer (Li-Cor; Lincoln, NE). Primary antibodies for U2AF65 (Abcam ab197031 with verified reactivity to human and rat) and JMJD6 (Abcam ab64575 with verified reactivity to rat and predicted reactivity to human) were diluted into Odyssey blocking buffer along with primary antibody to GAPDH (Abcam ab157156) loading control. These primary antibody solutions were made using a 1:1000 dilution of each. The box containing the membrane and antibody treatment was kept at 4°C overnight. The next morning, the membrane was washed three times with a TBS-T solution (1% TBS, 0.1% Tween). Secondary antibodies donkey-anti-rabbit (Li-Cor IRDye 680RD) and donkey-anti-goat (Li-Cor IRDye 800CW) were also diluted in Odyssey blocking buffer at a 1:15,000 concentration. The secondary was allowed to incubate for 45 min before again washing the membrane three times with 1× TBS-T. After washing, the membrane was imaged using a Chemidoc MP imager (BioRad; Hercules, CA) and analyzed utilizing ImageJ software (NIH). Western blot for media heparanase was also performed. Equivalent amounts of denatured media with 4× loading dye were loaded into each well of a stain-free gel and the protocol described above was followed, with the exception of HPA-1 primary antibody (HPA1 M-45 sc-25826) (Santa Cruz; Dallas, TX) having a 1:200 dilution.

ELISA: In order to measure the protein concentration in the media, an ELISA was performed for human sFlt-1. A DuoSet ELISA kit (R&D Systems, Cat #DY008, DY321B) was used in order to measure this protein. A blank 96-well plate was treated for 24 h with the appropriate capture antibody. After washing three times with 1× wash buffer, the plate was blocked with 1× reagent diluent for 1 h. The plate was again washed three times, and the standards and samples loaded with a 2-h incubation period. HUVEC media samples were diluted 1:40 with reagent diluent and BeWo samples were diluted 1:4. The plate was washed again, and the appropriate detection antibody was added to the plate for another 2 h. After washing the plate again, streptavidin-HRP was added the plate placed into a black box in order to offer protection from the light. After a 20-min incubation, the plate was washed again and the color reagent A and B combination was added to the plate and again protected from the light. After 20 min, the stop reagent was added and the plate was analyzed using the Infinite M200 Pro plate reader with associated Magellan software (Tecan; Grodig, Austria).

Statistical analysis: All statistical analyses were performed using the Prism 7 software (GraphPad). A Student’s *T*-test was used when two groups were compared, a one-way ANOVA was used to compare different groups for same end point, and a two-way ANOVA was used when multiple groups were assessed for various conditions. The Tukey post-hoc test was used in conjunction with these analyses and statistical significance was determined by a *P* < 0.05 between groups.

## Results

### Confirmation of U2AF65 and JMJD6 knockdown and overexpression

**Figure 1:**
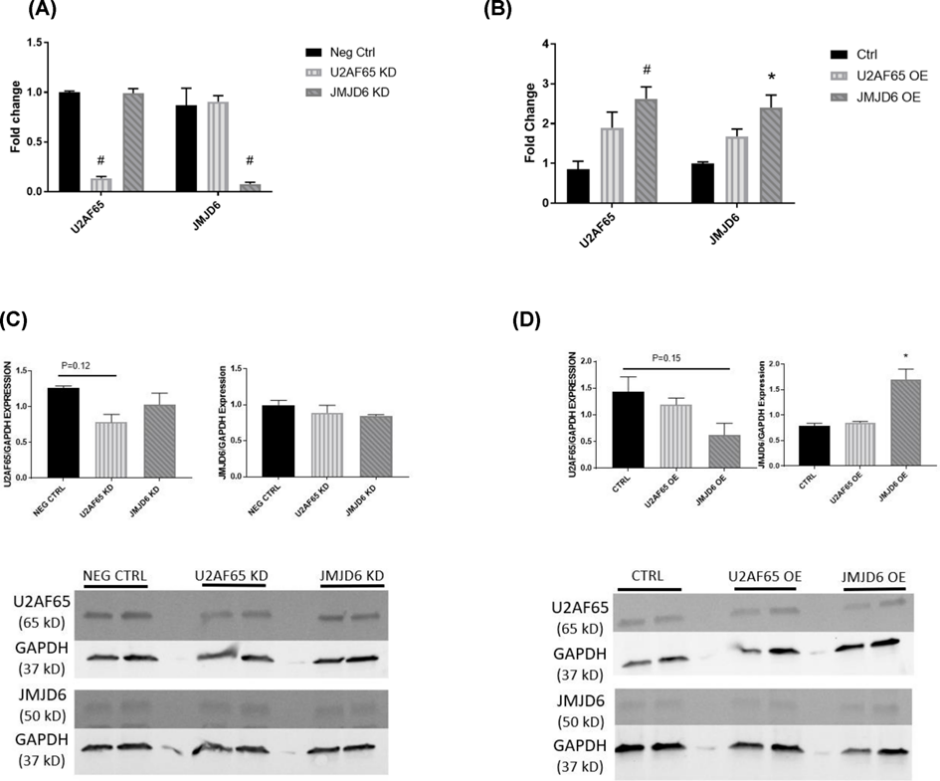
Confirmation of knockdown/overexpression in HUVECs rtPCR was performed to ensure that adequate knockdown (**A**) and overexpression (**B**) of U2AF65 and JMJD6 was achieved in HUVECs (*N* = 4). Western blots were also done to see if the message translated to the protein level. Western blots for knockdown of U2AF65 and JMJD6 are shown in (**C**) and samples from overexpression of the proteins are seen in (**D**) (*N* = 2). *P < 0.05; #*P* < 0.01

We first set out to examine the *in vitro* effects of U2AF65 and JMJD6 modulation on sFlt-1 splicing through siRNA knockdown and overexpression of both. In order to confirm that the siRNA knockdown and plasmid overexpression of U2AF65 and JMJD6 was successful, real-time PCR (rtPCR) was performed to assess the mRNA expression. HUVEC knockdown of U2AF65 resulted in a significant reduction such that expression was only 13.6% of control (1 ± 0.01 fold change vs. 0.136 ± 0.02 fold change, *P* < 0.001), and JMJD6 expression was not affected in these samples. HUVEC knockdown of JMJD6 also achieved a reduction of nearly 90% compared with control (0.87 ± 0.17 fold change vs. 0.077 ± 0.02 fold change, *P* < 0.001) (Figure 1A). Overexpression of U2AF65 in HUVECs, though not reaching significance, was more than double compared with control (0.85 ± 0.20 fold change vs. 1.90 ± 0.33 fold change, *P* = 0.11). JMJD6 was successfully upregulated compared to control (1.27 ± 0.27 fold change vs. 2.40 ± 0.31 fold change, *P* < 0.05). Interestingly, samples that were stimulated to up-regulate JMJD6 also exhibited a significant increase in U2AF65 compared with control (0.85 ± 0.20 fold change vs. 2.63 ± 0.30 fold change, *P* < 0.01) (Figure 1B). Western blots of both knockdown (Figure 1C) and overexpression (Figure 1D) were performed in order to determine the effect of both knockdown and overexpression. Surprisingly, there was no change in protein expression among any of the groups, knockdown or overexpression.

**Figure 2:**
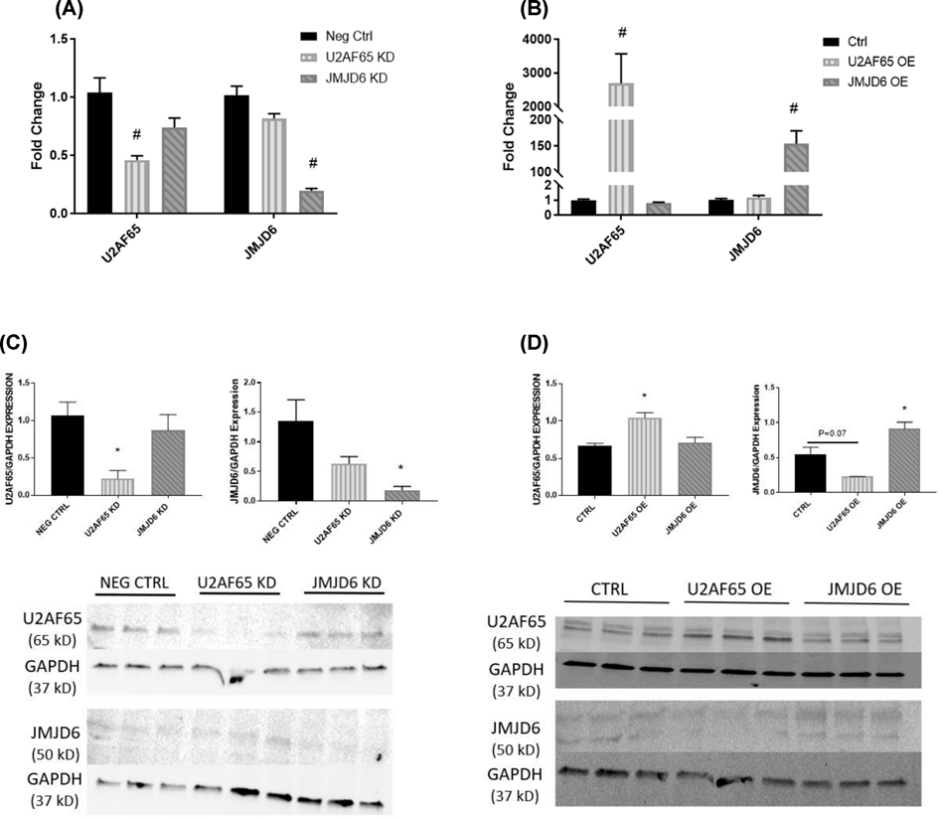
Confirmation of knockdown/overexpression in BeWo rtPCR was performed to ensure adequate knockdown (**A**) and overexpression (**B**) was achieved in BeWo placental trophoblasts (*N* = 6). Additionally, Western blots were performed to see if the message translated to the protein expression. U2AF65 and JMJD6 knockdown Western blots and their analyses are shown in (**C**), while overexpression can be observed in (**D**) (*N* = 3). *P < 0.05; #*P* < 0.01

Additionally, rtPCR was performed on samples from BeWo placental trophoblasts to confirm knockdown. U2AF65 was reduced 54% (1.04 ± 0.12 fold change vs. 0.46 ± 0.04 fold change, *P* < 0.001) and JMJD6 was reduced 80% compared with control (1.01 ± 0.08 fold change vs. 0.20 ± 0.02 fold change, *P* < 0.001). Similar to HUVEC, the knockdown of these proteins in BeWo did not have an impact on the other (Figure 2A). U2AF65 had a robust up-regulation in BeWo compared with control (1.01 ± 0.06 fold change vs. 2694 ± 878 fold change, *P* < 0.001) and no effect on JMJD6 expression. Overexpression of JMJD6 was also achieved (1.02 ± 0.09 fold change vs. 155 ± 24 fold change, *P* < 0.001) (Figure 2B). Again, Western blots were performed to examine protein levels of U2AF65 and JMJD6. Unlike HUVEC, BeWo knockdown of U2AF65 achieved a significant decrease in the protein compared with control (1.07 ± 0.18 vs. 0.22 ± 0.11 U2AF65/GAPDH expression; *P* < 0.05) and knockdown of JMJD6 was similarly decreased (1.34 ± 0.37 vs 0.18 ± 0.07 JMJD6/GAPDH expression; *P* < 0.05) (Figure 2C). Overexpression of U2AF65 achieved significant upregulation (0.67 ± 0.03 vs 1.04 ± 0.07 U2AF65/GAPDH expression; p<0.05) and JMJD6 overexpression also led to a significant increase in JMJD6 protein (0.55 ± 0.10 vs. 0.92 ± 0.09 JMJD6/GAPDH expression; *P* < 0.05). Interestingly, BeWo cells in which U2AF65 was overexpressed had a near-significant trend in JMJD6 reduction compared with control (0.55 ± 0.10 vs. 0.23 ± 0.002 JMJD6/GAPDH expression; *P* = 0.07) (Figure 2D).

### U2AF65 and JMJD6 effect on sFlt-1 splicing

**Figure 3:**
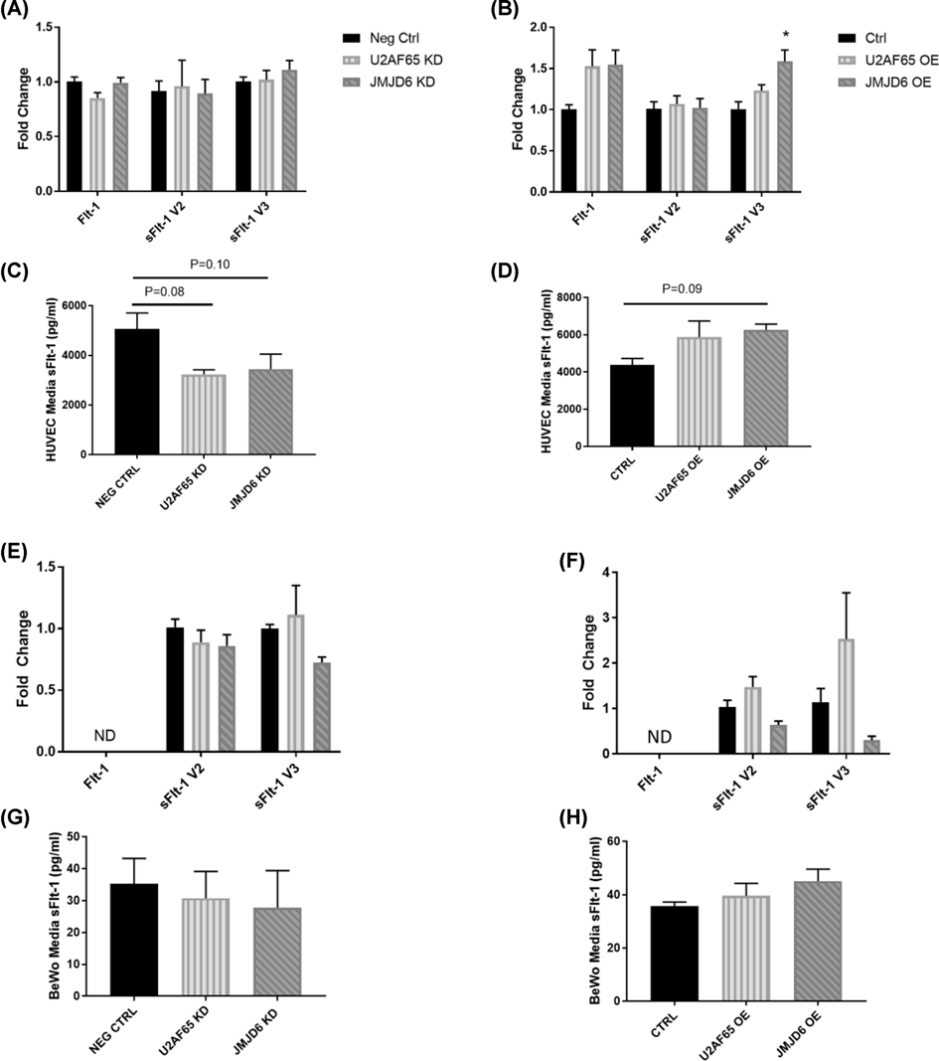
Effect of U2AF65 and JMJD6 expression on sFlt-1 HUVEC knockdown (**A**) and overexpression (**B**) samples were analyzed for impact on sFlt-1 mRNA, with JMJD6 overexpression causing significantly increased sFlt-1 version 3 expression (*N* = 4). Media from HUVEC samples were analyzed for sFlt-1 protein. Both knockdown (**C**) and overexpression (**D**) media showed trends of sFlt-1 protein in the same direction as U2AF65 and JMJD6 expression (*N* = 4). BeWo knockdown (**E**) or overexpression (**F**) had no effect on sFlt-1 mRNA (*N* = 6). Similarly, media collected from U2AF65 or JMJD6 knockdown (**G**) or overexpression (**H**) had no difference in media sFlt-1 concentration (*N* = 6). Two-way ANOVA was performed on the rtPCR samples, and one-way ANOVA was performed on media sFlt-1 samples *P < 0.05 vs. control. ND = Not detectable.

After confirming that the expression of U2AF65 and JMJD6 were knocked down, we next determined the effect of their modulation on FLT1 gene variants: Flt-1, sFlt-1 version 2 (V2), and sFlt-1 version 3 (V3). In both HUVEC and BeWo knockdown of U2AF65 and JMJD6, there was no effect on the expression of any variant at the mRNA level (Figure 3A,E). When protein from HUVEC knockdown media was measured for sFlt-1; however, there were trends for decreased sFlt-1 (5066 ± 644 pg/ml Neg Ctrl vs. 3231 ± 194 pg/ml U2AF65 KD, *P* = 0.08; 5066 ± 644 pg/ml Neg Ctrl vs. 3447 ± 605 pg/ml JMJD6 KD, *P* = 0.10) (Figure 3C). This decrease in media sFlt-1 was not observed in BeWo samples (Figure 3G). The FLT1 variants were also measured in cells overexpressing U2AF65 and JMJD6. While U2AF65 overexpression did not have an effect on sFlt-1 splicing in HUVECs or BeWos, JMJD6 overexpression led to significant increases in sFlt-1 V3 in HUVECs (1.01 ± 0.09 fold change Ctrl vs. 1.59 ± 0.14 fold change JMJD6 OE, *P* < 0.05) (Figure 3B,F). HUVEC media was also measured for sFlt-1, and we found that there was a near significant increase in media sFlt-1 in samples taken from JMJD6 overexpression, and trends for increased media sFlt-1 in samples from U2AF65 overexpression (Figure 3D). Similar to the knockdown media, media taken from BeWo overexpression samples had no change in sFlt-1 (Figure 3H).

### U2AF65 and JMJD6 on heparanase expression

**Figure 4:**
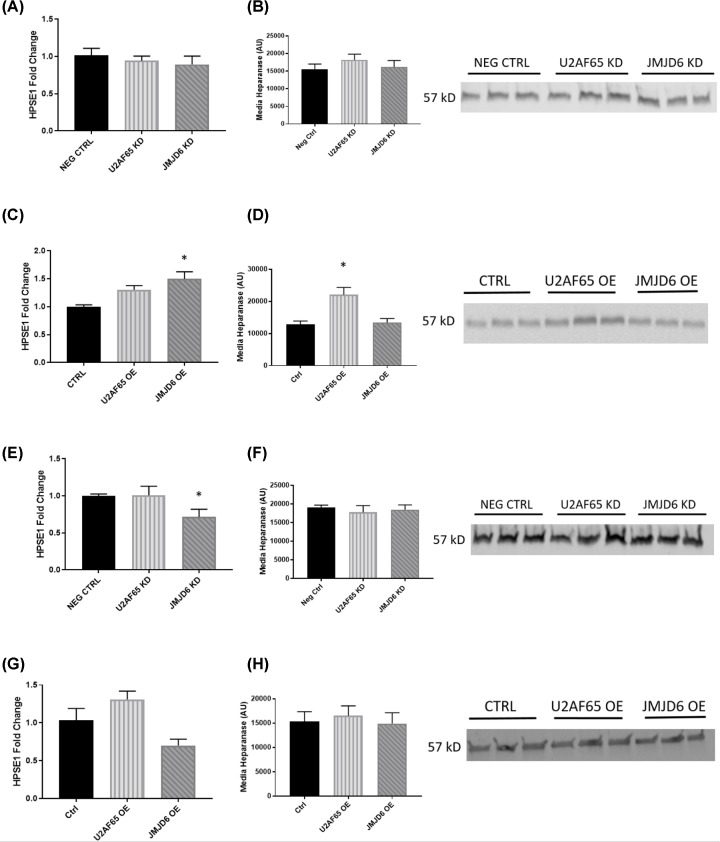
Effect of U2AF65 and JMJD6 expression on heparanase HUVEC knockdown samples showed that there was no effect on heparanase mRNA expression (*N* = 4) (**A**) or media protein (*N* = 3) (**B**). HUVEC JMJD6 overexpression had significant increase in heparanase mRNA (*N* = 4) (**C**), but only HUVEC overexpression of U2AF65 had significant increase in media heparanase protein (*N* = 3) (**D**). BeWo knockdown of JMJD6 showed significantly decreased heparanase message (*N* = 6) (**E**), but none of the BeWo knockdown groups showed any change in heparanase media protein (*N* = 3) (**F**). BeWo overexpression of U2AF65 and JMJD6 had no effect on heparanase mRNA (*N* = 6) (**G**) or media protein (*N* = 3) (**H**). One-way ANOVE was performed for all groups. *P < 0.05 vs. control.

We have previously reported that sFlt-1 mRNA was not changed in exposure to hypoxia. Extracellular matrix enzyme heparanase, however, was significantly increased [22]. Heparanase cleaves heparan sulfate chains on the extracellular matrix, allowing bound sFlt-1 to be released. Given the trends for sFlt-1 in the media to follow U2AF65 and JMJD6 expression, we decided to measure heparanase when expression of these proteins was altered. Though we found that heparanase expression did not change in HUVECs with decreased U2AF65 or JMJD6 expression (Figure 4A,B), there was a significant decrease in heparanase when JMJD6 expression was reduced in BeWos (1.00 ± 0.07 vs. 0.55 ± 0.11 fold change; *P* < 0.05) (Figure 4E). Western blot of media from these samples, though, showed that there was no change in heparanase protein (Figure 4F). While heparanase expression was not affected in HUVECs where U2AF65 or JMJD6 were decreased, overexpression of JMJD6 led to significant increases in heparanase (1.00 ± 0.03 vs. 1.50 ± 0.13 fold change; *P* < 0.05) (Figure 4C). Interestingly, the only samples with increased heparanase by western blot analysis were U2AF65 overexpression media samples (12,777 ± 1099 vs. 22,081 ± 1207 AU; *P* < 0.05) (Figure 4D). Conversely, overexpression of either U2AF65 or JMJD6 had no effect on heparanase expression in BeWo at mRNA or protein level (Figure 4G,H).

### Effect of hypoxia on U2AF65 and JMJD6 expression

**Figure 5:**
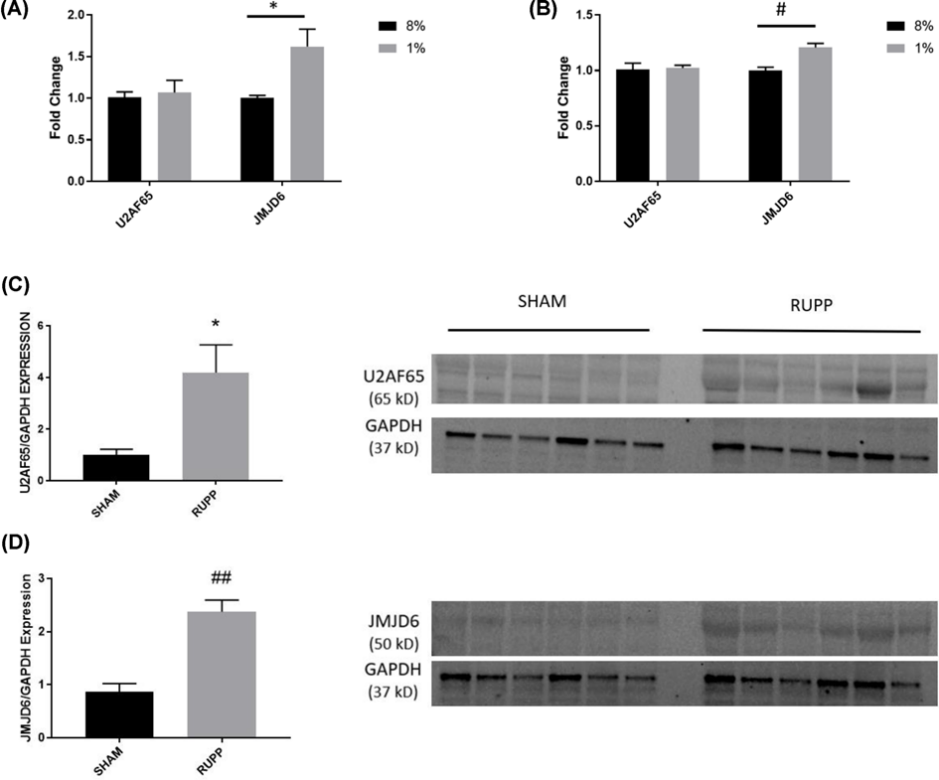
Hypoxia regulation of U2AF65 and JMJD6 HUVECs cultured in hypoxia for 24 h had significantly up-regulated JMJD6, with no change in U2AF65 expression (**A**). Similarly, BeWo cells exposed to hypoxia also had unchanged U2AF65 but significantly elevated JMJD6 (**B**). Chronic hypoxia of the RUPP placenta, however, had significantly increased U2AF65 (**C**) and JMJD6 (**D**) compared with sham animals. Two-way ANOVA was performed for both cell groups. Student’s *t*-test was performed on the placental protein data. *N* = 6 in all groups. *P < 0.05; #*P* < 0.01 ##*P* < 0.001 vs. control/ sham.

Because hypoxia is associated with increased release of sFlt-1, we exposed cells to 8% and 1% oxygen, to mimic the conditions of the normal and preeclamptic placenta, respectively, for 24 h. Though hypoxia had no effect on U2AF65 expression in either HUVEC or BeWo samples, JMJD6 was significantly up-regulated in both cell lines under hypoxic conditions (HUVEC: 1.00 ± 0.03 vs. 1.62 ± 0.21 fold change; *P* < 0.05) (BeWo: 1.03 ± 0.01 vs. 1.21 ± 0.04 fold change; *P* < 0.01) (Figure 5A,B). As stated above, we previously showed that hypoxia did not cause changes in sFlt-1 mRNA. The HUVEC samples, however, had a near-significant trend of increased sFlt-1 V3 expression, but did not have changes in expression of other FLT1 forms (data not shown).

### U2AF65 and JMJD6 are significantly increased in RUPP placentas

Due to the increase of JMJD6 in hypoxic cell culture, we set out to determine if this protein was increased by chronic ischemia *in vivo*, by utilizing the rodent RUPP model of placental ischemia-induced hypertension. Using placental protein from animals that underwent the sham or RUPP procedures, we found that JMJD6 was significantly increased in the placentas of RUPP animals compared with sham (0.87 ± 0.15 fold change vs. 2.40 ± 0.22 fold change; *P* < 0.001) (Figure 5D). Unlike the hypoxic exposure in cell culture, we also saw that U2AF65 was significantly increased in the placentas of these animals as well compared with sham (1.00 ± 0.22 vs. 4.19 ± 1.08 fold change; *P* < 0.05) (Figure 5C).

## Discussion

Abnormal production of sFlt-1 has been shown to be detrimental in pregnancy and plays an important role in the development of preeclampsia [4]. The mechanism governing the alternative splicing of sFlt-1, however, has been poorly understood. U2AF65 has been reported to be associated with the spliceosome for sFlt-1 [23]. Due to its interaction with U2AF65, JMJD6 has been believed to participate in the regulation of sFlt-1 splicing [18].

There are conflicting reports of the exact role that these proteins play in the splicing of sFlt-1. Boeckel et al. [23] demonstrated first that JMJD6 may play a role in sFlt-1 splicing regulation. Using siRNA knockdown of JMJD6, they found that sFlt-1 was significantly increased and endothelial tube formation was impaired [23]. Additionally, Palmer et al. [15] reported that JMJD6 expression was decreased in hypoxic cell culture as well as in preeclamptic placentas. They also saw that knockdown of JMJD6 was associated with increased sFlt-1 production. The group also proposed that U2AF65 promotes the splicing of sFlt-1, but JMJD6 hydroxylates lysine residues on U2AF65, which will ultimately lead to its degradation [15]. Alahari et al. [16], however, found that JMJD6 was significantly increased in hypoxia and the preeclamptic placenta. It was also up-regulated in placentas during early gestation, when oxygen levels are low [16].

**Figure 6:**
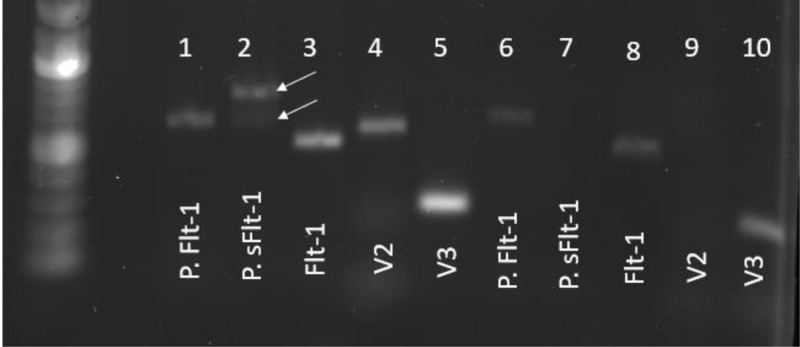
Validation of FLT1 primers BeWo and HUVEC samples were used to examine the validity of previously published and newly designed primers. The first five samples are from BeWo and in the following order: previously published Flt-1 (P. Flt-1), previously published sFlt-1 (P. sFlt-1), newly designed Flt-1 (Flt-1), newly designed sFlt-1 V2 (V2), newly designed sFlt-1 V3 (V3). The next five samples are from HUVEC and in the same order as BeWo. The previously published sFlt-1 appears to have multiple bands in BeWo samples, indicated by the white arrows, while it does not appear at all in HUVEC samples. HUVEC samples also do not seem to express much sFlt-1 V2, as our primer for it cannot be seen, despite having a clear band in BeWo samples.

Interestingly, in the present study, we arrived at slightly different results than that observed in some previous studies [15,23]. There are several potential sources of this discrepancy. For one, our study, as well as the earlier ones, relied on primary cells (HUVECs) which may be heterogeneous in their biological activity. The individual studies also utilized different primers for the various sFlt-1 isoforms (Figure 6), which may lead to differences in the resulting data. Additionally, earlier studies relied heavily on qRT-PCR to assess efficiency of knockdown of individual splicing proteins. In our hands, achievement of knockdown at the mRNA level did not necessarily translate to changes at the protein level, suggesting regulation of the proteins at the level of protein turnover and translational efficiency. However, differing methods of protein quantification between studies may have resulted in differences in reported knockdown efficiency. These are all confounding factors in comparing the present study with these earlier reports.

Yi et al. showed that JMJD6 and U2AF65 interact in an RNA-dependent fashion. They also found that 74% of splicing reactions by JMJD6 and 67% of splicing reactions by U2AF65 were regulated by both proteins. Of the total 383 splicing reactions that are co-regulated by U2AF65 and JMJD6, 77% were altered in the same direction [17]. Despite the idea that JMJD6 hydroxylation of U2AF65 leads to U2AF65 degradation mentioned above, the fact that these proteins frequently work together and in the same direction would contradict that hypothesis. Our own data also contradict the idea of JMJD6 leading to U2AF65 degradation, as overexpression of JMJD6 did not cause reduction of U2AF65 expression, and knockdown of JMJD6 did not lead to increased levels of U2AF65. Interestingly, overexpression of U2AF65 led to a near-significant decrease in JMJD6 protein expression in BeWo, suggesting U2AF65, or a downstream target of U2AF65, caused increased degradation of JMJD6.

Because we saw trends for sFlt-1 released into the media following the overexpression or knockdown patterns in spite of having little effect on mRNA levels, we decided to look at alternative mechanisms by which protein secretion could be altered post-translationally; namely the extracellular matrix enzyme heparanase. We have previously shown that in cultured BeWo cells, hypoxia leads to a significant increase in heparanase [22]. Heparan sulfate chains on extracellular proteins can bind sFlt-1 in its heparin binding site. The cleavage of these chains by heparanase allows for the bound sFlt-1 to be released into the media. Interestingly, we saw that knockdown had no effect on heparanase expression in HUVEC, but reduction of JMJD6 in BeWo samples led to significantly decreased heparanase (HPSE) mRNA expression. This decreased expression, however, did not appear to translate to the protein expression. Conversely, overexpression of JMJD6 did not have an effect on HPSE expression in BeWo samples, but a significant increase in mRNA was observed in HUVEC samples. Western blot analysis of HUVEC samples showed that only U2AF65 overexpression had increased heparanase expression. Though the reason for this is unknown, it may be important to note that HUVEC U2AF65 overexpression was only partially successful, resulting in moderate, non-significant increases in both U2AF65 and JMJD6.

Our studies show that U2AF65 and JMJD6 may not be necessary for sFlt-1 splicing, or JMJD6 may have a minor role in the splicing of sFlt-1 V3. It is also possible that the two merely promote the splicing event, and only one of them is necessary for splicing to continue. It also seems likely that JMJD6 has a role in heparanase expression, given the changes in JMJD6 expression led to changes in heparanase expression at the mRNA level. The different response to changes in U2AF65 and JMJD6 expression in the different cell types also begs the question about different regulation patterns of different tissues. JMJD6 is known to have a multitude of functions and is implicated in several cancers, however, to our knowledge, no one has examined the function of U2AF65 or JMJD6 in detail across different tissues. Therefore, future research should be aimed at better understanding how these proteins behave in various tissues and different environmental conditions in order to better understand their role in alternative splicing.

## Abbreviations

HPSE: heparanase
HUVEC: human umbilical vein endothelial cell
JMJD6: jumonji C-domain containing protein 6
PE: preeclampsia
sFlt-1: soluble VEGF receptor-1
U2AF65: U2 auxiliary factor 65
